# Using deformable registration to assess dosimetric impact of variability in deep inspiration breath hold (DIBH) levels for left breast treatment

**DOI:** 10.1002/acm2.70541

**Published:** 2026-04-10

**Authors:** Venketesh Thrithamara Ranganathan, Amy Gouthro, Tynan Stevens

**Affiliations:** ^1^ Department of Radiation Oncology Cape Breton Cancer Center Sydney Nova Scotia Canada

**Keywords:** BH target variability, deformable registration, DIBH, left‐sided breast dosimetry

## Abstract

**Background:**

In deep inspiration breath hold (DIBH) left breast radiation treatment, dosimetric impact of variation within breath hold (BH) tolerances has not been precisely quantified.

**Purpose:**

Estimate target and organs at risk (OAR) dose variations due to patient movements within BH target tolerances, and assess impacts on 3DCRT, VMAT and pseudo skin flash planning.

**Methods:**

Twenty patients who underwent left breast DIBH treatment with 4000 cGy in 15 fractions using 3DCRT or VMAT were retrospectively selected. A combination of deformable registration and breathing traces from CT simulation were used to generate CT volumes corresponding to ± 5 mm about the BH target. 3DCRT clinical treatment plans were applied directly to the generated CT volumes. For VMAT, new optimizations were performed on the target BH CT scan to achieve target dose within ± 0.5 % of the treated plan. The re‐optimized plan was applied to the generated CT volumes to estimate the organ doses. Pseudo skin flash treatment plans were created for the VMAT patients using the same optimization parameters, and applied to the generated CT volumes. Target coverage (D_95_), heart, lung, and breast mean doses, and heart hotspot (D1cc) were evaluated at each deviation.

**Results:**

Deformable registration demonstrated relative motion of the target and OARs not previously observed with simple rigid registration methods. Target coverage in 3DCRT plans were more robust against BH target variation compared to VMAT and pseudo skin flash. However, pseudo skin flash show comparable target coverage to 3DCRT for BH target overshoot. With 3DCRT, the heart hot spot shows significant individual variations in the undershoot regime.

**Conclusion:**

Deformable registration‐based dose estimation reveals complex organ motion with significant individual variability in plan robustness. 3DCRT is less sensitive to BH variability than VMAT. Our protocol potentially enables individualized approach to selecting technique and tolerances based on patients’ DIBH movements.

**Abbreviations**
DIBH: Deep inspiration breath hold
DIR: Deformable image registration
DVF: Deformation vector field

## 1 Introduction

Female breast cancer is the second most frequently diagnosed cancer, accounting for 11.6% of the total cancer cases globally, as per the Global Cancer Statistics 2022.^1^ Global estimates show that about 25% of children who became maternal orphans were due to female breast cancer incidence in 2020. ^2^ Breast cancer incidence and thus its consequences necessitate advancements in personalized cancer treatment. Radiotherapy remains the treatment management for two‐thirds of all cancers. For breast cancer patients, radiation treatment can either be performed postmastectomy or to the intact breast depending on the clinical factors.

Side effects of breast radiation could include erythema, microcapillary vascular damage, vascular atrophy, atelectasis, radiation pneumonitis, ^3^ lymphedema, reduced shoulder mobility, and brachial plexus neuropathy.^4^ Studies have shown a linear correlation between the global mean heart dose and ischemic cardiac incidence, and a 4–16% increase in major coronary incidence per Gy of heart mean dose ^5^, ^6^, ^7^, ^8^ Radiation exposure to other structures such as the left anterior descending artery is known to introduce risk of adverse cardiac events.^9^

Deep inspiration breath hold (DIBH) is a procedure in which patients hold their breath during radiation treatment, known to spare heart exposure in left breast treatment.^10^ The DIBH maneuver tends to increase the spacing between the heart and chest wall due to a combination of inferior motion of the heart and anterior motion of the chest wall, thus reducing heart exposure. However, individual patients may exhibit considerably different breath hold (BH) techniques and internal motion, the dosimetric impact of which has not previously been assessed. Following the widespread use of treatment techniques like 3‐dimensional conformal radiation therapy (3DCRT), intensity modulated radiation therapy (IMRT), and volumetric modulated arc therapy (VMAT), studies have explored the efficacy of these techniques in DIBH for heart sparing.^11^, ^12^, ^13^ While all these techniques are efficient in achieving the target dose, differences in the nature of the dose distributions produced may impact robustness to DIBH variations. For example, it is reported that while VMAT spares OARs from high dose, it increases the volume of low dose exposure.^12^

For the implementation of DIBH, patient breathing traces are routinely acquired during patient simulation to establish a BH target position. Tolerance margins about this target position are needed in practice as some amount of both residual motion during BH and variability in depth of BH are unavoidable. While there are no strict guidelines for surface monitoring based DIBH target tolerance,^14^, ^15^ a range of margins from +/− 1 mm to +/− 5 mm are clinically practiced. However, these clinical decisions are not always based on informed dosimetric knowledge of target and OARs, given the difficulty in modeling complex internal anatomical movements. Simple surface monitoring does not represent internal organ movements, and thus, dosimetric estimation of target and OARs based on surface monitoring alone remains insufficient.^14^ Current dosimetric knowledge on BH target variation is chiefly limited to models reflecting rigid motion only, often by simply shifting the plan isocenter position.^16^, ^17^ To the best of our knowledge, there are no studies that have reported the dosimetric impact at various BH target variations using a detailed model of internal motion.

In this work, we aim to employ a deformable image registration (DIR) approach to precisely model the complex motion of internal organs to capture the effects of DIBH variability in left breast treatment. We then estimate the dose to the target and OARs for three commonly used techniques for DIBH left breast treatment: 3DCRT, VMAT, and pseudo skin flash.^18^ We believe that our approach captures complex internal anatomical motion and the resulting dosimetry more accurately than simpler methods such as rigid image registration (RIR) and isocenter shifts for estimating intrafraction organ‐dose variation. This framework meaningfully informs DIBH tolerance margins, and potentially advances personalized radiotherapy by enabling individualized BH thresholds based on each patient's actual dosimetric impact to OARs and to target coverage.

## 2 METHODS

### 2.1 Patient selection

We retrospectively identified 20 patients with left‐sided breast cancer who had undergone DIBH radiotherapy to the whole breast, without nodal involvement. Patients were treated with either 3DCRT or VMAT. The inclusion criteria were restricted to patients treated to a total dose of 4000 cGy in 15 fractions using 6 MV photon beams for VMAT and a combination of 6MV/10MV/18MV for 3DCRT. Patients with insufficient anatomy included in their simulation scans for accurate deformable registration calculation were excluded.

### 2.2 Estimation of deformation vector fields (DVFs)

To characterize the complex anatomical motion during BH target variation in DIBH procedure, including anterior chest wall displacement and inferior abdominal motion, we used the Velocity AI software package (Varian Medical Systems, Palo Alto, CA). A novel protocol was developed to estimate tissue deformation across free‐breathing (FB) and BH states.

**FIGURE 1:**
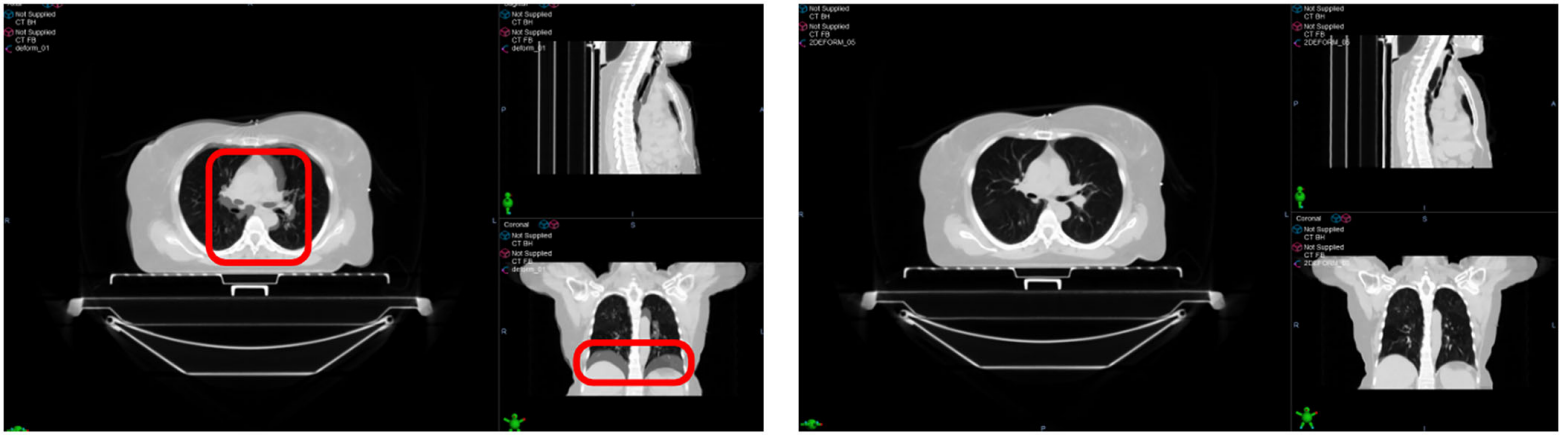
Left: Image co‐registration between FB and BH CT scan using deformable algorithm of velocity AI (red boxes highlight inferiority in image co‐registration using deformable algorithm). Right: Image co‐registration between FB and BH CT scan using extended deformable multi‐pass algorithm of velocity AI.

The DVFs, that consists of the displacement vector of every voxel pair was generated by co‐registering the FB and BH CT scans were generated using the scans acquired during the CT simulation. An extended deformable multi‐pass B‐spline registration algorithm was employed, as it has demonstrated superior performance compared with other DIR models.^19^ An example from our work is shown in Figure 1, where deformable algorithm of Velocity AI is seen to underperform the extended deformable multi‐pass algorithm. Manual refinement was performed for patients with large anatomical displacements by using sub‐regions of interest to improve local registration accuracy.

**FIGURE 2:**
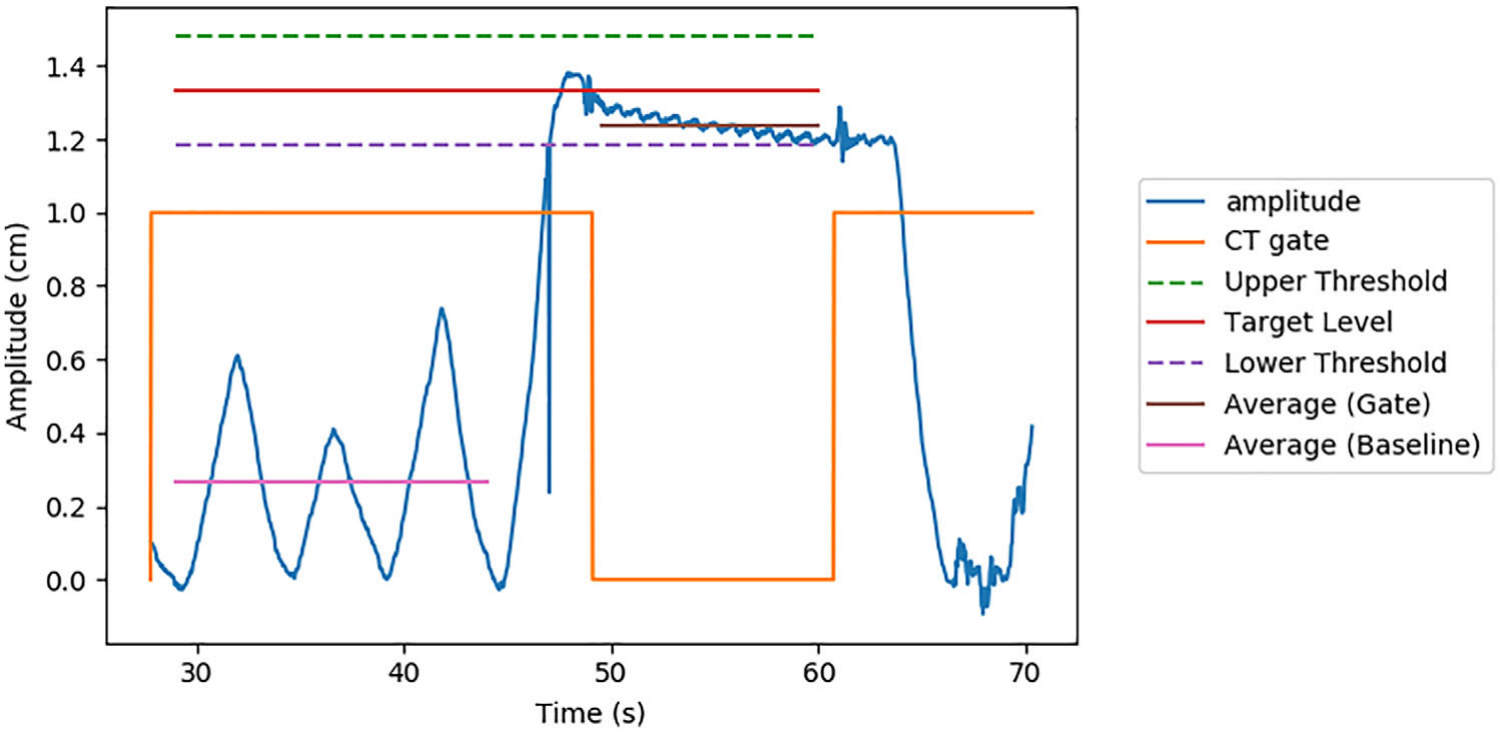
Representative patient breathing trace during CT simulation. Average values for FB and BH were computed. The amplitude difference (∆) between these averages was used as a scaling factor to generate DVFs corresponding to different BH deviations.

Respiratory traces were acquired during simulation using the Real‐time Position Management (RPM) or Respiratory Gating for Scanners (RGSC) system (Varian Medical Systems). The RPM reflector block was placed for each patient on the midline of the abdomen, at the position of greatest breathing motion. The same system was used for monitoring BH target motion during treatment. Average baseline and BH amplitudes were established from these traces (Figure 2). DVFs corresponding to deviations of ± *δ* mm from the BH target were calculated by applying a scaling factor:

(1) $$DVF_{\delta }=DVF_{0}*\left(1+\delta /\Delta \right)$$

where *δ* is the target deviation and ∆ represents the difference in amplitude between baseline and BH positions. For example, if ∆ = 10 mm, a 1 mm deviation from BH was represented by linearly scaling the DVF by 10%; if ∆ = 5 mm, the scaling factor was 20%. An in‐house Python program (Python v3.8, numpy v1.19.5) was used to generate scaled DVFs for deviations ranging from −5 mm to +5 mm in 1 mm increments.

### 2.3 Propagation of structure sets

Propagation of structure sets was performed to evaluate anatomy corresponding to each deviation. First, the BH structure set was propagated to FB images using Velocity AI, with FB designated as primary and BH as secondary. The scaled DVFs were then imported and applied to the FB images to generate resampled CT volumes for each deviation. Finally, structure sets were propagated from the FB images to these resampled CTs, designating the resampled CT as primary and the FB as secondary.

### 2.4 Evaluation of registration accuracy

To ensure registration accuracy, a dosimetric approach was employed. The PTV and heart dose metrics for the optimized plan on the original BH CT were compared against the same plan recalculated on the 0 mm deviation to assess the reliability of DVF‐based image generation. While small dosimetric errors are expected due to limitations of the registration algorithm, large errors should be avoided as they may indicate gross inaccuracies in the representation of the FB to BH motion by the DVF. We therefore considered patients with average percent difference between PTV_D95_ (0 mm) and PTV_D95_ (Clinical) less than 3 % and the percent difference between heart mean dose (0 mm) and heart mean dose (Clinical) less than 5 % eligible for our study.

### 2.5 Planning techniques

Patient contouring was performed on the DIBH scan, following the NRG published RADCOMP atlas for breast radiotherapy. PTV volumes for planning included the whole breast expanded by 5 mm isotropically, and this contour was retracted by 5 mm from the skin surface for dose evaluation. For VMAT, an optimized plan was created on the BH CT to achieve planning target volume (PTV) coverage within 0.5% of the clinically delivered dose. The same beam arrangement was used as in the clinical plan: 2–3 arcs of approximately 180–200 degrees arc length, typically from 10 to 30 degrees off posterior to 60 degrees past anterior, with exact placement driven by patient anatomy and planner discretion. Optimization objectives included upper and lower target objectives, and upper and mean objectives on the contralateral breast, ipsilateral lung and heart, with exact values and priorities driven by the original clinical planning process. This plan was then applied to the DVF‐generated CTs corresponding to deviations from −5 mm to +5 mm. For pseudo skin flash, virtual bolus of 10 mm thickness with −500 HU was applied that is expected to minimize the effects due to setup uncertainties.^20^ The same VMAT optimization parameters were used, and the optimizer was run in a single pass to generate dose distributions. As with VMAT, the pseudo skin flash treatment plan was applied to different deviations for the dose estimation. All VMAT patients were treated with 6 MV photons. For 3DCRT, the clinical forward planned tangent beam arrangements were used directly, with 2–10 segments per field, and a mixture of 6/10/18 MV as needed to achieve the desired dose distribution. All clinical treatment plans achieved PTV_D95_ of 95% or greater and were created using Eclipse v18.0 software by Varian. AcurosXB algorithm (v15.6) was used for dose calculations.

## 3 RESULTS

Table 1 shows average anatomical movements of the target and OARs for the largest observed deviation from the BH target (i.e. + 5 mm). +/− Z indicate inferior/superior motion and +/− Y represent anterior/posterior movements. Anterior‐inferior motion of the heart is the most pronounced, while the PTV and contralateral breast move oppositely in the superior direction, emphasizing the nonrigid nature of the BH motion. Overall, the heart executes a pronounced displacement over other structures.

**TABLE 1:** Organ displacements (average vector displacement) for the +5 mm BH deviation compared to the expected treatment position taken from the 0 mm deviation model.

Organ	*X*‐axis (mm)	*Y*‐axis (mm)	*Z*‐axis (mm)
PTV	0.7 ± 0.2	3.1 ± 0.5	−2.6 ± 0.4
Heart	2.0 ± 0.3	4.1 ± 0.4	6.2 ± 1.0
Lungs	0.8 ± 0.2	3.4 ± 0.4	3.4 ± 0.8
C.Breast	0.1 ± 0.2	2.9 ± 0.4	−3.0 ± 0.4

Over the range of RPM deviations tested, the DVF estimated lung volumes vary considerably. Averaged across the patient sample using relative lung volumes, the lung compresses by 14.4 ± 1.3 % at a BH target deviation of −5 mm and expands by 11.1 ± 1.0 % at +5 mm. The heart volumes showed the opposite trend, increasing by 3.3 ± 1.3 % in the undershoot extremum and about 3.4 ± 1.2 % contraction in the overshoot extremum. This latter result appears to reflect changes to the envelope of the pericardium during the BH maneuver, but some artificial component due to crosstalk with the rapidly changing adjacent lung volumes cannot be ruled out. In both cases, the volumes change linearly between these extremes, due to the linear interpolation used to predict the intermediate DVFs. The contralateral breast and PTV volumes were by contrast relatively independent of the BH target deviation.

Table 2 shows the percent differences between clinical plans and the 0 mm CT volume, which ideally corresponds to the actual BH target scan, for both PTV_D95_ and heart mean dose. The average percent difference for PTV_D95_ is −0.2 ± 0.1 cGy, and for heart mean dose it is −1.2 ± 0.4 cGy, indicating high dosimetric agreement between clinical and 0 mm plans. The dose agreement between the 0 mm and BH scan were slightly worse for the heart due to relatively large motion of this organ between BH and FB as inferred from table 1, nonetheless the registrations were inspected visually and found to be acceptable. We consider a heart mean dose difference less than 5 % eligible for the study.

**TABLE 2:** Comparison of PTV_D95_ and heart mean dose between 0 mm and clinical plans for 20 patients. The average percent difference between PTV_D95_ (0 mm) and PTV_D95_ (Clinical) is −0.2 ± 0.1 cGy, and the percent difference between heart mean dose (0 mm) and heart mean dose (Clinical) is −1.2 ± 0.4 cGy.

Patient	PTVD 95 (0 mm)	PTVD 95 (Clinical)	% Diff	Heart mean dose (0 mm)	Heart mean dose (Clinical)	% Diff
Patient 1	3877.3	3880.2	−0.1	86.5	87.9	−1.6
Patient 2	3850.8	3931.9	−2.1	56.6	58.6	−3.4
Patient 3	3815.1	3815.4	0.0	118.2	120.4	−1.8
Patient 4	3825.8	3847.6	−0.6	95.8	97.0	−1.2
Patient 5	3885.8	3878.2	0.2	99.4	102.2	−2.7
Patient 6	3878.9	3880.4	0.0	150.2	152.2	−1.3
Patient 7	3932.9	3929.6	0.1	89.9	93.1	−3.4
Patient 8	3959.5	3963.2	−0.1	77.7	78.1	−0.5
Patient 9	3861.3	3877.4	−0.4	160.7	167.7	−4.2
Patient 10	3911.2	3943.5	−0.8	80.0	78.6	1.8
Patient 11	4003.5	3999.7	0.1	205.1	213.1	−3.8
Patient 12	3974.0	3956.2	0.5	223.3	225.2	−0.8
Patient 13	3917.8	3916.7	0.0	229.0	227.8	0.5
Patient 14	3950.8	3929.3	0.5	269.6	272.4	−1.0
Patient 15	3908.7	3949.8	−1.0	277.8	277.1	0.3
Patient 16	3907.9	3899.9	0.2	271.8	275.6	−1.4
Patient 17	3958.3	3961.7	−0.1	274.5	267.9	2.5
Patient 18	3984.8	3982.6	0.1	215.7	219.7	−1.8
Patient 19	3914.5	3914.3	0.0	270.8	267.6	1.2
Patient 20	3953.4	3946.3	0.2	276.1	281.5	−1.9

**FIGURE 3:**
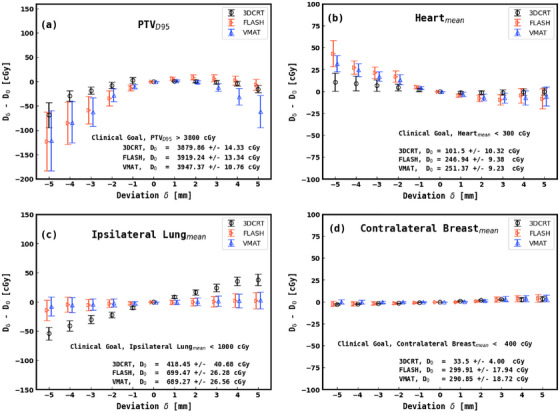
Dose difference at BH deviations from −5 mm to +5 mm relative to the 0 mm reference for (a) PTV_D95_, (b) heart mean dose, (c) ipsilateral lung mean dose, and (d) contralateral breast mean dose. A 0.1 cm horizontal offset is applied to the VMAT curve for visual clarity.

Figure 3(a) shows the dose differences in PTV_D95_ for BH target deviations ranging from −5 to +5 mm relative to the 0 mm reference. In the overshoot regime (0 to +5 mm), PTV_D95_ remains relatively stable for 3DCRT and pseudo skin flash, while VMAT fails to meet the optimization dose objective beyond +3 mm. In the undershoot regime (0 to −5 mm), PTV_D95_ consistently falls below the optimization goal of 3800 cGy for deviations exceeding −3 mm. At −5 mm, VMAT and pseudo skin flash show reductions of approximately 120 cGy, whereas 3DCRT exhibits a smaller reduction of about 70 cGy. All three modalities meet planning goals within the −2 mm to +2 mm range. Figure 3(b) shows that mean heart dose is largely independent of BH target variation for 3DCRT. However, in the undershoot regime, both VMAT and pseudo skin flash exhibit dose increases. At 0 mm, heart doses from VMAT and pseudo skin flash are approximately double those from 3DCRT. For ipsilateral lung (Figure 3(c)), 3DCRT shows a linear dose increase with overshoot, while VMAT and pseudo skin flash demonstrate minimal sensitivity to BH variation. In all techniques, contralateral breast dose is largely invariant with BH deviation (Figure 3(d)), although 3DCRT delivers nearly ten times lower dose than VMAT and pseudo skin flash.

**FIGURE 4:**
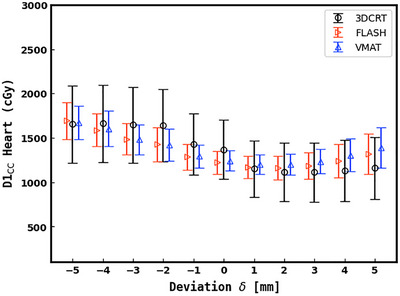
Heart D1cc versus BH target deviation. Large error bars for 3DCRT indicates the presence of high variability in dose estimation for individual patients from the mean D1cc.

**FIGURE 5:**
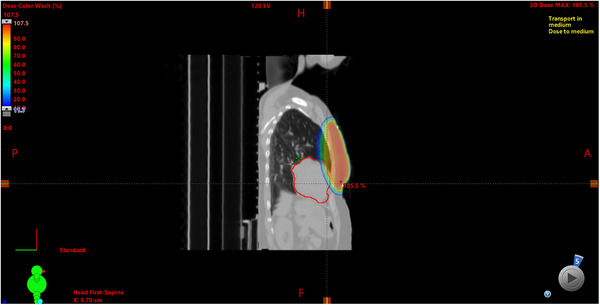
Example 3DCRT plan at −5 mm deviation. The anterior‐inferior portion of the heart enters the high‐dose region, significantly increasing D1cc.

**FIGURE 6:**
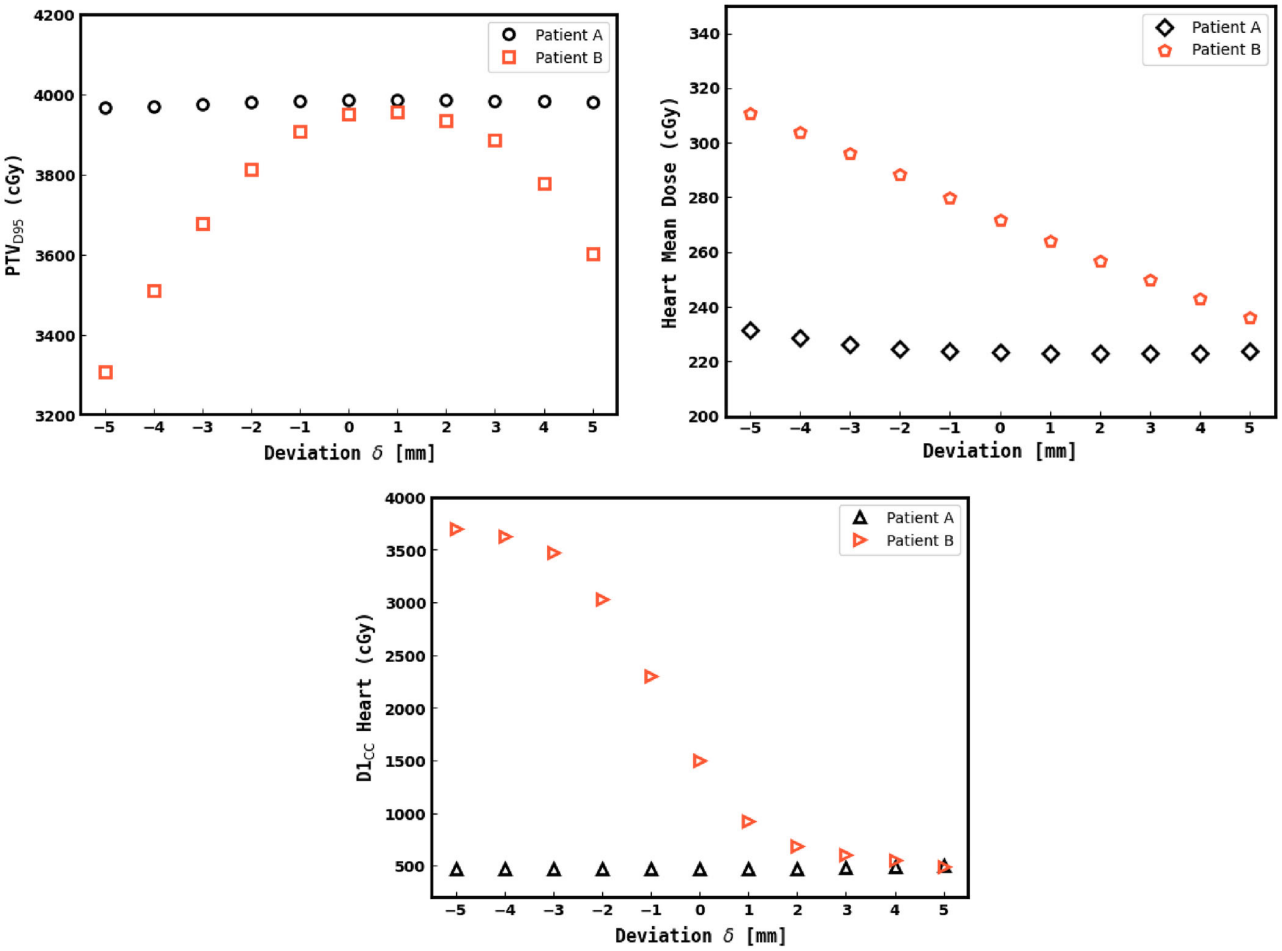
Example plots for dose versus BH target deviation for patients independent of target deviation (Patient A) and sensitive to BH target deviation (Patient B).

Figure 4 presents heart D1cc data, showing minimal dependence on BH overshoot for all techniques, and comparable increases between techniques for undershoot. However, 3DCRT exhibits greater variability especially in the undershoot regime. A large part of this variability may be explained by the sharp dose gradient at the posterior border with tangent techniques and whether or not the heart crosses into this region for a given patient. Figure 5 shows a patient who falls into this latter category, and Figure 6 demonstrates individual dose versus DIBH deviation plots for two patients at either extreme in terms of dosimetric sensitivity to motion. From this it can be seen that individual variability is impactful for both target and OAR dosimetry. For example, one such case shows D1cc rising from 1622.0 cGy at 0 mm to 3704.6 cGy at −5 mm—well above the clinical limit of 2000 cGy. Similarly, an individual patient observation showed a PTV_D95_ drop from 3950.8 cGy at 0 mm to 3307.3 cGy at −5 mm was observed, despite the average change for VMAT at this deviation being approximately 120 cGy (as seen in Figure 3(a)), highlighting the potential for clinically significant underdosing. Pseudo skin flash planning had less variability than VMAT between patients in the overshoot regime, owing to the extra MLC margin in the anterior and lateral directions.

## 4 DISCUSSION

Accurate dose delivery to the PTV while minimizing exposure to surrounding OARs is a central goal in radiotherapy. This is particularly challenging in left‐sided breast cancer treatments using DIBH when the lung inflation state varies due to patient compliance resulting in BH target variability. Approaches such as cone‐beam CT to planning CT registration^21^ to understand the heart position variability in a DIBH left breast treatment provides a snapshot of anatomy variation at treatment initiation to planning CT but cannot systematically investigate deviations from the BH target. As well as, an RIR approach cannot capture the interfraction dose variations precisely owing to the nonrigid motion of the organs. To our knowledge, no previous study has evaluated the dosimetric consequences of BH target deviations using realistic deformable anatomy changes within the clinically accepted tolerance of ± 5 mm for left‐sided DIBH.

It should be noted that our procedure analyzed only systematic errors and modeled them as constant deviations from BH targets for the duration of treatment. In a realistic treatment scenario, intra‐fraction variation in BH levels and drift within individual BHs will present a more complex scenario. Using real patient BH traces from treatment and weighted sums of the static deviations assessed by our method could approximate these scenarios, but is outside the scope of this study. The static deviations assessed here can be viewed as a worst case scenario for a given level of clinical DIBH target margins (i.e. if using ± 3 mm margins, the worst case scenario is a patient is at the margin threshold for their whole treatment). This analysis also implicitly assumes that there is no concurrent systematic error in patient positioning (i.e. that during IGRT the patient was setup to the correct position, and that deviations during treatment are due to varying patient performance of the DIBH).

In this work, we developed and implemented a protocol using Velocity AI to generate CT volumes at multiple BH target deviations from −5 mm to +5 mm. To validate these generated volumes, we compared the clinical plan with the corresponding 0 mm deviation plan, showing excellent dosimetric agreement and confirming the overall robustness of the registration and CT generation protocol. Some residual errors were observed qualitatively around where the diaphragm meets the chest wall, as well as around the inferior border of the heart. From table 1, it is clear that the heart undergoes relatively large motion compared to the other structures, likely contributing to the slightly worse co‐registration accuracy in this region compared to the region around the PTV. It should also be noted that the study implicitly assumes that linear scaling of the DVF will accurately reflect the internal motion during DIBH overshoot and undershoot. This assumption is relatively sound for small displacements from the target, but may worsen for large displacements. Nonetheless, we believe this DIR based model is more reflective of the complex anatomical motions during DIBH maneuver than simple rigid registration techniques.

Gnerucci et al. ^16^ report intrafraction dosimetric variation in a DIBH left breast treatment by estimating the dose metrics for isocenter shifted treatment plans. For 3DCRT technique, they report a higher variation for OARs of small volume consistent with our observation. However, their analysis indicate that the variations did not exceed 3 Gy, while our results show that individual patients may exceed 3 Gy at clinically practiced tolerance extremum for 3DCRT. For the ipsilateral lung, 3DCRT shows a linear dose increase with overshoot, but this does not present a major clinical concern. In contrast, heart dose increases by an average of 0.5 Gy in the undershoot regime for VMAT and pseudo skin flash. All three techniques show minimal sensitivity of contralateral breast and ipsilateral lung doses to BH target deviation. Without real‐time BH monitoring, up to 22% of BHs may exceed the ± 5 mm threshold,^22^ increasing the risk of such events.

Kugele et al. ^17^ also used isocenter shifts to estimate dosimetric impact of DIBH variation in tangent treatments, and found that PTV coverage decreased most for isocenter shifts in the left/anterior/cranial directions as this shifts the PTV towards the smaller posterior and medial margins. This is consistent with our observation of larger effects in undershoot than overshoot for tangent based fields. They noted the opposite was observed for OARs, with right/posterior/caudal isocenter shifts being more impactful as they brought OARs into the treatment field. For example, heart D2% and lung *V*_20_ *_Gy_* reported to increase by approximately 10–15%, 20–25% and 60–70% for posterior isocenter shifts of approximately 1, 3, and 5 mm respectively. We observed much smaller increases to lung mean dose, and no consistent increases to heart mean or hotspot doses in the overshoot regime, which we interpret as the impact of the more realistic DIR based analysis. Specifically, with DIR we see that the increase to in field lung tissue is partially offset by the overall increase to lung volume, and the heart moving inferiorly in over inspiration prevents it from moving in‐field due to the anterior displacement. In fact, our observation was that heart doses were more impacted by undershoot of the DIBH target, a result that is not seen with simple isocenter shift methods.

Our DIR based approach to evaluating dosimetric variability in DIBH can be used to guide institutional acceptable threshold windows specific to a given planning technique and dosimetric tolerances. Importantly, this approach could also be used to allow for the DIBH target tolerances to be set on an individual basis. For instance, patients with BH difficulty may qualify for a BH treatment given the dose objectives are met at clinical tolerance extremum of +/− 5 mm, and receive benefit of the BH procedure. On the other hand, there might be patients with underdosed PTV or higher OAR dose if a tighter tolerance is not insisted upon. Referring to Figure 6, it is clear that the dose profiles similar to patient A can have less BH tolerance constraint over the patients whose dose profile resemble that of patient B. Thus, we strongly believe that our DIR protocol for organ dose estimation at different BH target tolerances advances the potential of personalized precision radiation treatment.

## 5 CONCLUSION

The delivery of high‐quality, personalized radiotherapy requires precise knowledge of target and OAR doses. The use of DIBH technique for left breast radiotherapy is intended to improve sparing of OARs by increasing the lung volume and the heart to chest wall separation. While image guidance ensures the patient matches their planned position immediately before treatment and surface monitoring is widely used to monitor breath‐hold reproducibility, residual BH variation within the DIBH target tolerances during treatment remains. Furthermore, the motion observed at the patient surface during DIBH monitoring can differ in both magnitude and direction from internal OAR motion, and the dosimetric impact of the resulting complex anatomical relationships have not previously been accurately evaluated.

In this study, we developed a protocol for modeling tissue motion under varying BH target deviations in left‐sided breast DIBH, demonstrating its utility in identifying both target underdosing and unexpected OAR hot spots. Importantly, this work represents a major step forward compared to rigid translation techniques in terms of accurately representing the complex anatomical motion involved in DIBH and its impact on dosimetry. This approach may be used to inform both institutional level and individualized DIBH tolerance limits, in a way that is sensitive to both the treatment technique, dose distribution, and patient anatomy. In future work, we intend to explore ways to further streamline the process for identifying patients and plans that are more robust or sensitive to DIBH variability, as well as exploring methods to increase the general robustness of treatment planning techniques to DIBH variability.

## AUTHOR CONTRIBUTIONS

VTR and AG Contributed to data analysis, project execution, and manuscript preparation, TS contributed to project design, data analysis, project execution, and manuscript preparation

## CONFLICT OF INTEREST STATEMENT

The authors declare no conflicts of interest.
